# Chemical Characterization and Biological Activities Evaluation of *Myrtus communis* L. Essential Oil Extraction By-Product towards Circular Economy and Sustainability

**DOI:** 10.3390/foods13142211

**Published:** 2024-07-13

**Authors:** Meriem Abdessemed, Saoussen Bouacida, Mohamed Turki, Hayet Ben Haj Koubaier, Souha Omrani, Radia Allouache, Nabiha Bouzouita, Romdhane Karoui, Ahmed Snoussi

**Affiliations:** 1 Laboratory of Innovation and Valorization for a Sustainable Food Industry, Higher School of Food Industries of Tunis, University of Carthage, LR21AGR04, 58 Avenue Alain Savary, Tunis 1003, Tunisia; abdessemed.meriem2016@gmail.com (M.A.); bouacidasaoussen1405@gmail.com (S.B.); mohamedturki21@gmail.com (M.T.); hayet.kbaier@esiat.u-carthage.tn (H.B.H.K.); omranisouha@yahoo.fr (S.O.); allouacheradia216@gmail.com (R.A.); bouzouita.nabiha@gmail.com (N.B.); 2 University of Artois, University of Lille, University of Littoral Côte d’Opale, University of Picardie Jules Verne, University of Liège, INRAE, JUNIA, UMR-T 1158, BioEcoAgro, F-62300 Lens, France; romdhane.karoui@univ-artois.fr

**Keywords:** *Myrtus communis* L., leaves, by-product, essential oil, phenolic compounds, antioxidant activity, antibacterial activity, anti-α-amylase activity

## Abstract

Essential oil (EO) extraction is a widespread practice generating huge amounts of solid plant by-products a potential source of bioactive compounds, on the one hand, and a detrimental risk for the environment that needs to be carefully considered on the other hand. The present study aims to valorize *Myrtus communis* L. leaf by-products obtained following EO extraction using a steam distillation unit through the recovery of phenolic compounds and the evaluation of their biological activities. The total phenols, flavonoids, and proanthocyanidins contents of the ethanolic extract by-product were higher than the control (leaves without extraction of EO). Their amounts increased from 69.30 to 88.06 mg GAE/g for total phenols, from 36.31 to 70.97 mg QE for flavonoids and from 19.74 to 21.49 mg CE/g of extract for proanthocyanidins. The identification of phenolic compounds by high-performance liquid-chromatography equipped with a reversed-phase (RP-HPLC) system revealed that the by-product sample includes more gallic acid, catechin, syringic acid and luteolin 7-O-glucoside but less p-coumaric acid and kaempferol than the control. Moreover, the mid-infrared spectroscopy (MIR) showed the presence of benzene ring characteristic of phenolic compounds at 756 cm^−1^, esters of aromatic acids and stretching vibrations of polyphenols at 1141–1234 cm^−1^, C=C stretching present in phenolic acids such as coumaric acid and catechin at 1604 cm^−1^. The assessment of antioxidant activity revealed that the ABTS^+•^ radical scavenging activity was significantly increased, whereas the DPPH^•^ radical inhibition activity and the ferric reduction antioxidant power were significantly decreased. The results indicated, as well, that *Myrtus communis* L. leaf by-products maintained a considerable antibacterial activity depending on the tested bacterial strain. Additionally, the anti-α-amylase activity was higher for the *Myrtus communis* L. leaf by-product extract. Therefore, *Myrtus communis* L. leaf by-products of EO extraction offer phenolic compounds with significant biological activities, contributing to the sustainable development and the promotion of circular economy by the recovery of valuable inputs from plant by-products.

## 1. Introduction

The circular economy is a novel strategy aiming to reduce resource inputs, wastes, by-products, energy losses and emissions by slowing down, closing and restricting material and energy circuits through improved and effective design, maintenance, repair, reuse, durable regeneration, renovation and recycling. The regenerative approach was recently developed by the European community to enhance competitiveness, protect businesses against resource scarcity, and promote the emergence of new markets and opportunities [1]. Managing waste in the agri-food sector is widely recognized as a crucial aspect of the circular economy approach, with direct economic, environmental, and social implications. This method provides a feasible way to manage the impacts generated in this sector by proposing actions and solutions allowing the reintegration of wastes and by-products into the production processes [2].

EO extraction from medicinal and aromatic plants is an important practice in the agri-food sector [3]. In fact, essential oils have various applications. They are used as flavor enhancers to improve the taste of processed foods and as natural preservatives to substitute commonly used synthetic molecules that are responsible for harmful health effects, such as butylhydroxylanisol (BHA), which can cause deafness, hyperactivity, asthma, increased blood cholesterol, liver damage and cancer, or butylhydroxyltoluene (BHT), which can lead to skin reactions, reproductive and blood problems, and cancer [4,5].

Nevertheless, EOs are generated in low amounts, with yields around 8% of the dry plant biomass. Therefore, considerable amounts of solid by-products and residues are generated, leading to environmental and economic detrimental risks that require attention [6]. Various techniques have been implemented to promote the use of plant by-products. The recovery of molecules of interest endowed with biological activities seems to be an alternative for their valorization.

In recent years, there has been a significant increase in published studies on the extraction and identification of bioactive compounds from solid plant by-products of distillation [6]. Research studies suggest that the by-products of EO extraction contain significant levels of non-volatile and heat-resistant substances belonging to various groups of phenolic compounds [3]. These compounds have a variety of beneficial biological properties such as antioxidant, anti-inflammatory, antimicrobial, anti-atherosclerotic, anti-diabetic, anti-allergic, and anti-mutagenic effects [7].

Skendi et al. [8] are among the scientists who have analyzed the phytochemical components of various plant by-products. They found that the total content of phenolic compounds and flavonoids in *Satureja hortensis* and *Salvia officinalis* increased after EO extraction. The researchers attributed this increase to the elimination of essential oils and other water-soluble constituents in the distillation residue and the degradation of cell walls by dehydration due to the heat treatment of steam distillation, which improves the extractability of phenolic substances bound to constituents of plant cell walls such as cellulose, hemicellulose, lignin, pectin and stem structural proteins.

*Myrtus communis* L. (*M. communis*), commonly known as myrtle, is a very interesting plant as it has widespread distribution throughout the Mediterranean region and is known to produce EO of high economic importance with various uses in the pharmaceutical, cosmetic, and agri-food industries [9]. Furthermore, the plant is recognized for its digestive, antiseptic, antimicrobial, astringent, and toning properties. Its bioactive compounds, including terpenes, phenolic acids, flavonoids, and anthocyanidins in various plant organs, contribute to its hemagglutinating, anti-hyperglycemic, and anti-inflammatory effects [10].

*M. communis* is a plant that has aroused a lot of interest among scientists. Several studies have been carried out to investigate the composition and the biological potential of the EO of different plant parts such as leaves [11], flowers [12], and floral buds [13]. Other researchers focused on the polyphenols fraction of leaves and fruits and their valorization into food formulation mainly for liquor preparation [14,15]. Moreover, the effectiveness of solvents, extraction methods and drying processes on bioactive phenolic compounds in myrtle leaf extracts have been investigated [16]. However, no studies have been conducted to ascertain the phenolic compounds profile and the biological activities of *M. communis* EO extraction by-product.

Therefore, the utmost objective of this study is to investigate the effect of steam distillation on the phenolic content and biological activities of *M. communis* leaf by-products. For this purpose, the contents of total phenols, flavonoids and proanthocyanidins were determined and the phenolic profile was analyzed using the RP-HPLC system and MIR technique. Furthermore, the antioxidant activity was evaluated by ABTS^+•^ and DPPH^•^ radical scavenging assays and FRAP test, as well as the antibacterial and anti-α-amylase activities.

The results obtained from this study will constitute a significant contribution to promoting the sustainable development of the medicinal and aromatic plant industry through the implementation of a circular economy and the recovery of novel inputs from these resources.

## 2. Materials and Methods

### 2.1. Chemical Reagents

The chemical reagents used in the study include Folin–Ciocalteu reagent, anhydrous sodium carbonate (Na_2_CO_3_), hexahydrate aluminium chloride (AlCl_3_, 6H_2_O), vanillin, hydrochloric acid (HCl), formic acid, 2,2-diphenyl-1-picrylhydrazyl (DPPH), 2,2′-azino-bis (3-ethylbenzthiazoline-6-sulfonic acid) (ABTS), potassium ferrocyanide (K_3_Fe(CN_6_)), Ferric chloride (FeCl_3_), ascorbic acid, gallic acid, quercetin, catechin, trichloroacetic acid, phosphate buffer (pH 7.2), 3,5-dinitrosalicylic acid (DNSA), and α-amylase enzyme were procured from Sigma-Aldrich Chemie (Steinheim, Germany). Acarbose was manufactured by Bayer Pharma AG (Darmstadt, Germany). Methanol and ethanol were obtained from Merck (Darmstadt, Germany).

### 2.2. Characterization of Phenolic Compounds

The leaves of *M. communis* were analyzed for their phenolic compounds by the determination of the total phenol, flavonoid, and proanthocyanidin contents, using spectrophotometric methods. For this purpose, ethanolic extracts (EEs) were prepared from the by-products of essential oil extraction as well as from control leaves (leaves not subjected to EO extraction). Furthermore, RP-HPLC analysis and MIR measurements were conducted.

#### 2.2.1. Ethanolic Extracts Preparation

The leaf samples of *M. communis* used in this study were collected from the north-west of Tunisia (Ain Drahem, Jendouba) in January 2021. They were identified botanically following the Tunisian flora and a voucher specimen was stored in the herbarium of the Higher School of Food Industries of Tunisia (ESIAT) [17].

The collected plant material was first cleaned to remove impurities and dried using a microwave oven following the procedure adopted by Snoussi et al. [8]. After that, myrtle leaves were separated into two (02) batches: the first one was considered as a control for the comparative study and the second one was subjected to an extraction of the EO fraction. EO extraction was carried out at 100 °C for 4 h, using a steam distillation apparatus, until there was no significant increase in the amount of collected EO. Then, the leaves obtained as by-products were collected, dried under the same conditions as the control (reaching a moisture content of 10%), and conserved until further analysis.

The ethanolic extracts of *M. communis* leaves were obtained by agitated maceration at room temperature. Briefly, 10 g of plant material was mixed with ethanol in water solution (80:20, *v*/*v*) for 72 h, with a change of solvent every 24 h (3 × 100 mL). The obtained extracts were combined, filtered, and concentrated under reduced pressure [16].

#### 2.2.2. Determination of Total Phenolic, Flavonoid and Proanthocyanidin Contents

The total phenol content was determined following the Folin–Ciocalteu colorimetric method [16]. In total, 500 µL of Folin–Ciocalteu reagent (diluted 10 times) and 1 mL of distilled water were added to 100 µL of the diluted ethanolic extract. The mixture was left for one minute, and then 1.5 mL of Na_2_CO_3_ aqueous solution (20%) was added. After two hours of incubation at room temperature in the dark, the absorbance was measured at 760 nm with a spectrophotometer (JENWAY 6705 UV/Vis, Stone, Staffs, UK). The results were reported as mg gallic acid equivalent per g of extract (mg GAE/g).

The total flavonoid amount was quantified using the aluminum chloride (AlCl_3_) colorimetric method [16]. An equal volume (1.5 mL) of the diluted extract and AlCl_3_, 6H_2_O ethanolic solution (2%) was mixed. After 10 min, the absorbance was read at 367.5 nm. The results were expressed in mg quercetin equivalent per g of extract (mg QE/g).

The proanthocyanidins level was estimated using an acid method [18]. 3 mL of 4% vanillin and 1.5 mL of HCl were added to 500 μL of the diluted extract and kept for 15 min at room temperature in the dark. The absorbance was read at 500 nm by a spectrophotometer. The results were expressed as mg of catechin equivalent per g of extract (mg CE/g).

#### 2.2.3. RP_HPLC Analysis

The phenolic compounds profile was determined using an RP_HPLC system (Agilent technologies 1260, Germany, Japan), which was equipped with a reversed-phase column (100 mm × 4.6 mm id × 3.5 μm particle size; Zorbax Eclipse XDB C18) from Agilent (Waldbronn, Germany), as well as with a UV diode-array-detector (200–400 nm). The experimental protocol was followed as described by Jdey et al. [19]. In brief, a volume sample (3 µL) was injected into the HPLC system under a flow rate of 0.4 mL·min^−1^. The mobile phase consists of two solvents: methanol and Milli-Q water (0.1% formic acid). Optimized gradient elution was performed as follows: 0–5 min, 10–20% A; 5–10 min, 20–30% A; 10–15 min, 30–50% A; 15–20 min, 50–70% A; 20–25 min, 70–90% A; 25–30 min, 90–50% A; 30–35 min, return to initial conditions. Phenolic compounds were identified by comparing their retention time to standards at 254 nm.

#### 2.2.4. Mid Infrared Spectroscopy Measurements

The spectra were recorded at room temperature (20 °C) between 4000 and 400 cm^−1^ at a resolution of 4 cm^−1^ with a Fourier transform spectrometer IRTracer-100 (Shimadzu, Duisburg, Germany) which was mounted with an attenuated total reflection (ATR) accessory equipped with a grip (Pike Technologies, Inc., Madison, WI, USA). The ATR cell was made with a diamond crystal. Before each measurement, the spectrum of the diamond crystal was recorded and used as background. Between different sample measurements, the crystal was carefully cleaned using ethanol and ultra-pure water.

### 2.3. Evaluation of Antioxidant Activity

The in vitro evaluation of the antioxidant capacity of *M. communis* leaf EEs was performed using three methods: the ABTS^+•^ and the DPPH^•^ radical scavenging assays and the FRAP test. Ascorbic acid was used as standard.

#### 2.3.1. ABTS^+•^ Radical Scavenging Assay

The ABTS^+•^ radical inhibition activity of EEs was assessed following the slightly modified protocol described by Sarr et al. [20]. More precisely, 1 mL of the extract, at different concentrations, was added to 1 mL of the freshly prepared ABTS^+•^ solution. The mixture was incubated for two min in darkness at room temperature, and the absorbance was read at 734 nm using a spectrophotometer (JENWAY 6705 UV/Vis, Stone, Staffs, UK). The radical scavenging activity (RSA) was determined using the following formula: RSA = ((1 − AE/AC) × 100), where AE represents the absorbance of the tested extract and AC represents the absorbance of the control sample (extraction solvent solution). Results were expressed as the concentration required to inhibit 50% of the ABTS^+•^ radical (IC_50_), deduced graphically.

#### 2.3.2. DPPH^•^ Radical Scavenging Assay

The DPPH^•^ radical scavenging assay was conducted following the protocol of Snoussi et al. [16], with some modifications. In total, 1 mL of the extract solution diluted to different concentrations was mixed with 1 mL of a methanolic solution of DPPH^•^ (2 × 10^−4^ M) and incubated for 30 min at room temperature in the dark. The absorbance of the mixture was measured at 517 nm using a spectrophotometer (JENWAY 6705 UV/Vis, Stone, Staffs, UK). The radical scavenging activity of DPPH^•^ and the IC_50_ was determined as indicated for the ABTS^+•^ radical scavenging test.

#### 2.3.3. FRAP Test

The FRAP test was conducted following the experimental protocol outlined by Gülçin et al. [21]. 2.5 mL of phosphate-buffered solution (pH 7.2) and 2.5 mL of 1% [K_3_Fe(CN_6_)] solution were introduced into test tubes containing 1 mL of diluted ethanolic extract at different concentrations and then incubated at 50 °C for 20 min. Afterward, 2.5 mL of 10% trichloroacetic acid was added. Subsequently, 2.5 mL of distilled water and 0.5 mL of 0.1% FeCl_3_ were added successively to the 2.5 mL previously removed from the mixture. The absorbance was then measured at 700 nm on a spectrophotometer (JENWAY 6705 UV/Vis, Stone, Staffs, UK). The blank assay was prepared under the same experimental conditions by replacing the extract by an 80% ethanolic solution. Results were expressed as the 50% effective concentration (IC_50_) at which the optical density was 0.5.

### 2.4. Evaluation of Antibacterial Activity

The antibacterial activity of natural substances is related to the functional groups of their main compounds (alcohols, phenols, terpenes and ketones) and their synergistic effects, as well as to the bacterial-tested strain [22].

The bactericidal activity of the studied EEs was estimated using four pathogenic bacterial strains namely: *Staphylococcus aureus* (ATCC 25923), *Listeria monocytogenes* (ATCC 19115), *Aeromonas hydrophila* (ATCC 49140) and *Salmonella enteritidis* (ATCC 13076) following the well-diffusion method reported by Bouhdid et al. [23]. The estimation scale of the antibacterial activity was provided by Mutai et al. [24]. Briefly, Petri dishes containing nutrient agar were inoculated with 106–107 CFU/mL of bacteria. Then, 5 mm diameter wells were made, and 50 µL of diluted extract solution (10 mg/mL) was poured into each well. After incubation at 37 °C for 24 h, the diameters of the inhibition zone (mm) were measured using a precision rule.

### 2.5. Evaluation of Anti-α-Amylase Activity

The amylase inhibitory activity of *M. communis* leaf EEs was evaluated following the protocol outlined by Thalapaneni et al. [25] by using starch as a substrate and acarbose as a standard. 200 µL of diluted extract or diluted acarbose was added to 200 µL of the pre-made enzyme solution by diluting 3 mg of lyophilized enzyme in 10 mL of phosphate buffer (pH 7.2) and then incubated at 25 °C for 10 min. Next, 200 μL of 1% strength solution was added, and incubation was performed at 25 °C for 15 min. Samples were then boiled for 8 min and then placed in an ice-cold water bath after the addition of 200 μL of DNSA to each mixture. Finally, 1 mL of distilled water was added so that their absorbance could be read at 540 nm using a spectrophotometer (JENWAY 6705 UV/Vis, Stone, Staffs, UK) against a blank test. The inhibition of α-amylase percentage (IP) of extract or acarbose relative to control was then determined as follows: IP (%) = ((AC − AS)/AC) × 100), where AC and AS were the absorbances of the control (extraction solvent solution) and sample, respectively. Data were expressed as IC_50_, which represents the concentration of inhibitor required to reduce enzyme activity by 50%.

### 2.6. Statistical Analysis

Statistical analysis was performed with IBM SPSS Statistics software, version 22 (IBM Corp., Armonk, NY, USA) using a 1-factor ANOVA test to visualize significant differences between samples with a confidence interval (*p* < 0.05). The obtained data were expressed as means of triplicate ± standard deviation.

## 3. Results and Discussion

The recovery of phenolic compounds from EO extraction by-products is an innovative practice to which more intention must be given. In fact, new inputs possessing various virtues can be generated. Interestingly, compounds extracted from distillation by-products can be employed in various fields, offering thus cheaper inputs and a contribution to the international efforts and global trends aspiring to protect the environment and to reduce the carbon footprint, on the other hand.

### 3.1. Total Phenol, Flavonoid and Proanthocyanidin Contents in M. communis Leaf Ethanolic Extracts

**Figure 1:**
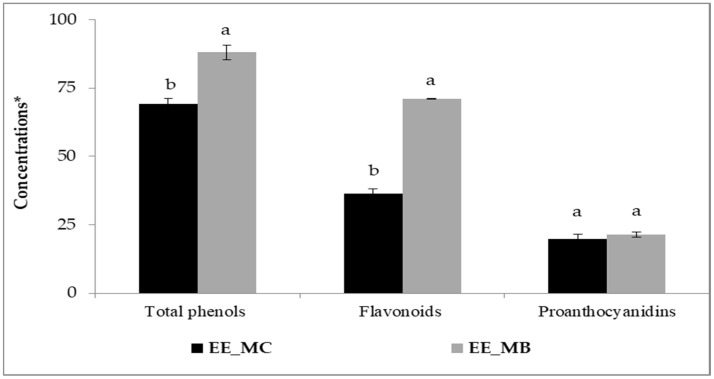
Total phenols, flavonoids and proanthocyanidins in ethanolic extracts of *M. communis* leaf control (EE_MC) and by-products (EE_MB). Total phenols were expressed in mg GAE/g, flavonoids were expressed in mg QE/g, and proanthocyanidins were expressed in mg CE/g. EE_MC: Ethanolic extract obtained from *M. communis* leaf control, EE_MB: Ethanolic extract obtained from *M. communis* leaf by-products. Each bar is the mean of 3 independent trials ± S.D. The mean concentrations values of total phenols (mg GAE/g), flavonoids (mg QE/g), and proanthocyanidins (mg CE/g) of the same sample followed by different lowercase superscript letters are significantly different (*p* < 0.05).

The process of EO extraction has significantly affected the amounts of phenolic compounds in *M. communis* leaves. Indeed, higher amounts of total phenols, flavonoids and proanthocyanidins were obtained for the extract prepared from the leaf by-products in comparison with the control (Figure 1).

Initially, the amounts of total phenols, flavonoids and proanthocyanidins were about 69.30 mg GAE/g; 36.31 mg QE/g and 19.74 mg CE/g, respectively. The obtained amounts of the studied extracts are higher than those reported by other researchers [16,26,27]. Snoussi et al. [16] found that the amounts of total phenols, flavonoids and proanthocyanidins for extracts obtained from *M. communis* leaves and dried using microwave oven were around 55.2 mg GAE/g, 28.2 mg QE/g and 10.5 mg CE/g, respectively. Several factors can influence the phenolic content of plant materials, primarily the plant species [28], the growth stage and the environmental factors (soil type, growing conditions, temperature) [16,26], the plant organs used [16,29], the drying process applied to raw materials [16], the solvent and extraction technique [16,27,30], as well as the methods and chemical reagents used in the analysis [16,26,31].

After EO extraction using steam distillation, the contents of total phenols, flavonoids of *M. communis* leaves were significantly increased to 88.06 mg GAE/g and 70.97 mg QE/g, respectively (*p* < 0.01). However, the content of proanthocyanidins showed no significant increase, reaching 21.49 mg CE/g (*p* > 0.05). It is worth mentioning that the flavonoid content in myrtle leaf by-products was almost twice higher than in the control. Our results are in good agreement with those obtained by Skendi et al. [8], who supported an increase in phenolic content after EO extraction by steam distillation from some plant materials. However, it has been found that the steam distillation process reduces the content of these compounds in the by-products. In their study, Christaki et al. [32] showed that the crude extracts of *Salvia rosmarinus*, *Salvia fruticosa* and *Mentha spicata* have higher total phenolics and flavonoids levels than the by-products of steam distillation and hydrodistillation, whereas the by-products of microwave-assisted hydrodistillation exhibited similar levels. They suggested that the distillation method and parameters may influence the phenolic compounds present in the distillation residues.

### 3.2. RP_HPLC Analysis of M. communis Leaf Ethanolic Extracts

The phenolic compounds present in *M. communis* control and by-product leaf ethanolic extracts were analyzed by RP-HPLC. Six compounds were identified and quantified based on their retention times and standard calibration curves.

Following the extraction of EO, the amounts of gallic acid, catechin, syringic acid, and luteolin 7-O-glucoside have heightened from 5.75 mg/g, 50 mg/g, 0.4 mg/g, and 11 mg/g to 12.7 mg/g, 77.4 mg/g, 4.45 mg/g, and 26 mg/g, respectively, while, p-coumaric acid and kaempferol contents were decreased from 25.2 mg/g and 0.9 mg/g to 15.7 mg/g and 0.3 mg/g, respectively (Table 1).

Numerous studies have proved that myrtle leaves comprise phenolic acids (caffeic, ellagic, syringic, vanillic, ferulic and gallic acids), flavonoids (myricetin and its derivatives, quercetin and its derivatives) and proanthocyanidins (catechin and its derivatives) [16,33,34,35].

In regard to the phenolic profile of EO extraction by-products from medicinal and aromatic plants, previous studies have consistently shown that these biomasses are rich in phenolic components with varying levels depending on several parameters, mainly the distillation technique and the chemical reactions that can occur during distillation, such as hydrolysis, thermolysis, and oxidation [3], as well as the subsequent treatment in the phenolic extraction process [36].

The obtained results revealed that the heat applied during steam distillation for EO extraction influences the content of phenolic compounds. Indeed, many studies focused on the effect of heat conditions on the stability of phenolics and they highlighted that it depends on their chemical structure [33]. Steam distillation is one of the most applied techniques for EO isolation during which the plant material is subjected to heating using dry steam at a temperature reaching 100 °C resulting in the release of volatile compounds, their training within a clarifier and their separation following a condensation step based on their immiscibility and density.

As a consequence, the structure of the cell wall of the biomass may be affected leading to a higher exposure of phenolic compounds to the heat and its subsequent effect. Golmakani et al. [37] demonstrated some changes in thyme leaf structure after hydro-distillation and microwave-assisted hydro-distillation procedures using scanning electron microscopy.

The increase in some phenolic compounds could be explained by the disruption of the integrity of cellular structure and their release from their combination to polysaccharides or protein bounds. However, the decrease in other compounds is mainly due to their sensitivity to heat and degradation. Some phenolic compounds are more vulnerable to degradation at high temperatures.

Among the degraded compounds, kaempferol content decreased by 67% after the EO extraction. The vulnerability of this compound is probably attributed, on one hand, to the presence of hydroxyl moiety in the B-ring which reduces the stability of flavonols and the absence of glycosylation on the hydroxyl group of flavonols significantly enhanced their stability, on the other hand [38].

Regarding coumaric acid, the estimated decrease is about 37%. Other researchers showed that the degradation mechanism of coumaric acid takes place under high temperatures and light [39].

### 3.3. Infrared Spectroscopy Measurements of M. communis Leaves and Ethanolic Extracts

In this part of the study, powders and ethanolic extracts of *M. communis* control and by-product leaves were analyzed by MIR spectroscopy to elucidate the structural changes following steam distillation of the phenolic fraction included in the plant material. Indeed, infrared spectroscopy is an analytical technique witnessing great interest among scientists and presents many advantages compared to conventional ones. In addition to being simple, fast, accurate, ecological, and cheaper than conventional techniques, it provides valuable information about specific absorption bands generated by vibrations between the bonds of different molecular groups in a sample [40].

**Figure 2:**
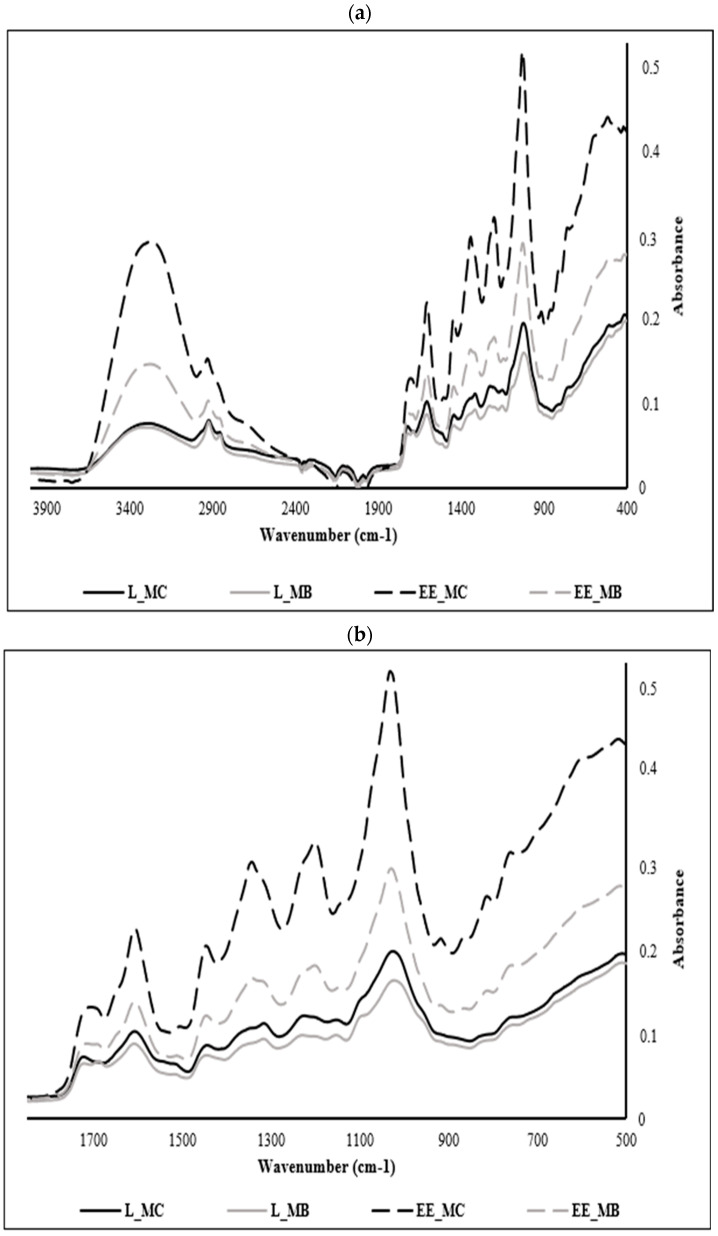
Mid-infrared spectra registered of powders and ethanolic extracts of *M. communis* leaf control and by-product: (**a**) Mid-infrared spectra registered between 4000 and 400 cm^−1^; (**b**) Mid-infrared spectra registered between 1700 and 500 cm^−1^. L_MC: *M. communis* control leaf powder, L_MB: *M. communis* by-product leaf powder, EE_MC: Ethanolic extract obtained from *M. communis* leaf control, EE_MB: Ethanolic extract obtained from *M. communis* leaf by-products. Each bar is the mean of 3 independent trials ± S.D.

*M. communis* samples were analyzed over the whole spectral range between 4000 and 400 cm^−1^ (Figure 2a); however, the interest in the phenolic compounds entailed us to focus on their specific region ranging from 1700 to 500 cm^−1^, considered as the fingerprint region of polyphenols in most of MIR spectra (Figure 2b).

The analysis of the different samples shows a similarity of several peaks but at varying absorbances, a sign of the variation in the concentration of phenolic compounds in each extract. The spectra of myrtle leaf powders (control and byproduct) show the least absorbance compared to those of ethanolic extracts since extraction is a process of concentration of these components. In the same context, we notice the appearance of new peaks in the spectra of the ethanolic extracts at 846, 910 and 1342 cm^−1^.

EE_MC spectrum is richer in phenolic compounds when comparing it with the ethanolic extract from leaf by-products (EE_MB). This phenomenon is explained by the sensitivity of polyphenols and their dependence on several parameters such as pretreatments of the plant matrix, extraction temperature, type of solvent, treatment duration and extraction conditions [41].

The peaks recorded at 756 cm^−1^ are attributed to the benzene ring characteristic of phenolic compounds and the peaks recorded at 1195, 1141 and 1234 cm^−1^ are ascribed to esters of aromatic acids (C-O-C) and stretching vibrations of phenolic components (C-OH) [42]. The peaks recorded at 810 and 864 cm^−1^ are assigned to the C-H deformations of polyphenols [43,44].

The region of the spectrum located between 864 and 1342 cm^−1^ is assigned to the major myrtle leaves monoterpenes, namely α-pinene at 864 cm^−1^ and 1,8 cineole at 1026, 1033, 1141, 1203, 1234, 1319 and 1342 cm^−1^. This result was found by Dhouibi et al. [11] who identified these compounds in the EOs of myrtle and rosemary with slight deviations. Likewise, Lopes et al. [45] confirmed that the region between 1050 and 1300 cm^−1^ corresponds to the OH, COO or C-O-C vibrations characteristic of polyphenols, flavonoids and tannins. In another research work, the peak observed at 1033 cm^−1^ in EE_MC spectra is assigned to the symmetric stretching observed in the C-O groups of flavonoids [46].

The band obtained at 1604 cm^−1^ can be attributed to C=C stretching present in the aromatic rings of polyphenols [47] such as coumaric acid [48]. In another research study, this band is attributed to aromatic structures bearing O-H groups in the ortho-position such as pyrogallol and gallic acid [49].

The peak observed at 1442 cm^−1^ is associated with the bending vibrations in the C-H groups of flavonoids [46], while that at 1712 cm^−1^ can be associated with certain hydroxy benzoic acids which present bands between 1715 and 1680 cm^−1^ with arylcarboxilic acid monomers [47] or might indicate the presence of flavonols [50,51] such as catechin [52].

For the overall spectra, the peaks recorded at 3302, 2924, and 2870 cm^−1^ are attributed to OH stretch vibration bands, CH asymmetric stretching, and CH symmetric stretching vibration bands, respectively [53] and the peak recorded at 2121 cm^−1^ corresponds to the C≡C stretch [54].

### 3.4. Antioxidant Activity of M. communis Leaf Ethanolic Extracts

The antioxidant activity of *M. communis* control and by-product leaf ethanolic extracts was evaluated using ABTS^+•^ and the DPPH^•^ radical scavenging assays and the FRAP test, and the results are presented in Table 2.

In consideration of the fact that the IC_50_ values are inversely correlated to the antioxidant activity, which indicates that higher IC_50_ means lower antioxidant activity, the ABTS^+•^ radical scavenging activity was significantly increased (*p* < 0.01), while the DPPH^•^ radical scavenging activity and the ferric reducing power was significantly decreased (*p* < 0.05), Indeed, after EO extraction, it decreased from 4 µg/mL to 2 µg/mL for the ABTS^+•^ test, slightly increased from 12 µg/mL to 13 µg/mL for the DPPH^•^ test and raised from 117 µg/mL to 163 µg/mL for the FRAP test.

Upon comparing the IC_50_ values of the three tests for *M. communis* leaf EEs with those of ascorbic acid, it was found that the ABTS^+•^ radical scavenging of the extracts was lower than that of ascorbic acid with a value of 16 µg/mL. However, it is notable that the DPPH^•^ radical scavenging IC_50_ values and the FRAP IC_50_ values were higher than that of ascorbic acid, with IC_50_ values of approximately 5 µg/mL and 92 µg/mL, respectively.

The ABTS^+•^ and DPPH^•^ radical scavenging IC_50_ values obtained for the *M. communis* control leaf extract were higher than those reported by Snoussi et al. [16] (2.4 µg/mL and 2.7 µg/mL, respectively). Similarly, for the FRAP test, Bouyahya et al. [55] indicated that *M. communis* leaf extract has a strong ferric-reducing activity and reported a lower IC_50_ value (41.97 µg/mL).

Regarding the evaluation of the antioxidant activity of EO extraction by-products from medicinal and aromatic plants using the ABTS^+•^ and DPPH^•^ radical scavenging tests and the FRAP test, our findings were in agreement with those of Skendi et al. [8], who reported that satureja, salvia and rosemary raw material extracts were less active against ABTS^+•^ radical and had lower ferric reducing power compared to the by-product extract.

Meanwhile, other scientists reported that the ABTS^+•^ radical scavenging activity of medicinal and aromatic plant extracts decreased after EO extraction. Among them, Christaki et al. [32] found that the raw material extracts of the tested materials had a significantly higher ABTS^+•^ inhibitory power than the by-product extracts obtained from the distillation, and they attributed the reduction in antioxidant activity to the distillation method and conditions, which may lead to the degradation of bioactive compounds and the loss of some molecules.

Méndez-Tovar et al. [56] and Chizzola et al. [57], indicated that the leaf extracts of the raw material are characterized by higher DPPH^•^ radical scavenging activity and FRAP values compared to the extracts of the by-products, explaining the decrease in the antioxidant activity by the removal of water-soluble compounds and essential oil from the raw plant material, which contribute to the total antioxidant activity.

### 3.5. Antibacterial Activity of M. communis Leaf Ethanolic Extracts

Differences were observed in the antibacterial activity of the tested extracts (Table 3). The results indicate a significant reduction in the inhibition zone diameter values of *M. communis* leaf by-products EE for *Salmonella enteritidis* (*p* < 0.05), *Aeromonas hydrophila* (*p* < 0.01) and *Listeria monocytogenes* (*p* < 0.05). Nevertheless, the inhibition zone diameter observed for *Staphyloccocus aureus* remains comparable for both extracts. Additionally, it can be noted that *Aeromonas hydrophila* and *Listeria monocytogenes* are the most sensitive Gram-negative bacteria, notably in response to the unextracted *M. communis* leaf extract. *Staphyloccocus aureus*, on the other hand, exhibited a similar inhibition zone diameter with both extracts, approximately 20 mm.

Several authors have evaluated the antibacterial activity of myrtle extracts on different bacterial strains [58,59,60].

According to Skendi et al. [61], distillation by-products may have potent antibacterial properties due to their abundance of phenolic compounds.

In general, Gram-positive bacteria are less sensitive compared to Gram-negative bacteria. This is attributed to the complex cell envelope of Gram-negative bacteria, which contains lipopolysaccharides that reduce the diffusion of hydrophobic antimicrobial molecules [62].

### 3.6. Anti-α-Amylase Activity of M. communis Leaf Ethanolic Extracts

**Figure 3:**
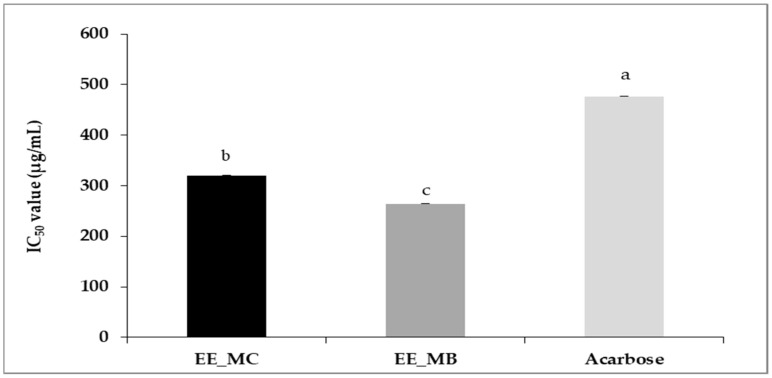
IC_50_ values of anti-α-amylase activity (μg/mL) of ethanolic extracts of *M. communis* leaf control (EE_MC) and by-product (EE_MB). EE_MC: Ethanolic extract obtained from *M. communis* leaf control; EE_MB: Ethanolic extract obtained from *M. communis* leaf by-products. Values are the mean of 3 independent trials ± S.D. The mean IC_50_ values of anti-α-amylase activity (μg/mL) of samples followed by different lowercase superscript letters are significantly different (*p* < 0.05).

The ethanolic extract obtained from *M. communis* control leaves was characterized by an α-amylase inhibitory potency significantly lower than that of leaf by-products ethanolic extract, with IC_50_ values of 320 ± 0.01 µg/mL and 264 ± 0.00 µg/mL, respectively (*p* < 0.01). Compared to acarbose (IC_50_ = 477 ± 0.01 µg/mL), the studied extracts showed a higher potency to inhibit α-amylase activity (Figure 3).

Recently, Al-Maharik et al. [63] reported a higher IC_50_ value for α-amylase inhibition activity of *M. communis* from Jenin approximately 795.43 ± 1.88 µg/mL. Indeed, the possibility of using plants in the treatment of diabetes has been previously studied and it has been demonstrated that their α-amylase inhibitory power is related to their phenolic composition, such as flavonoids, tannins and terpenoids [64,65].

## 4. Conclusions

The findings of this study showed that the *M. communis* leaf by-products had high total phenolic, flavonoid and proanthocyanidin contents. RP-HPLC analysis revealed that the by-product contained high amounts of gallic acid, catechin, syringic acid and luteolin 7-O-glucoside. Furthermore, mid-infrared spectroscopy measurements indicated the presence of benzene rings characteristic of phenolic compounds, esters of aromatic acids, stretching vibrations of polyphenols, and stretching vibrations present in phenolic acids such as coumaric acid and catechin.

The evaluation of biological activities showed that *M. communis* leaf by-products have a relevant antioxidant activity, allowing them a potential natural antioxidant source for preserving the quality of various products against oxidation, such as fatty foods. Additionally, they demonstrated an interesting ability to inhibit alpha-amylase, suggesting they could be used as an alternative to acarbose in the treatment of diabetes. Moreover, it was found that they have the potential to reduce the development of *Salmonella enteritidis*, *Aeromonas hydrophila* and *Listeria monocytogenes* strains.

Thus, the present study revealed that the by-product from *M. communis* leaves of EO extraction contains significant levels of phenolic substances endowed with considerable biological activity. This promotes circular economy and sustainable development through the valorization of bioactive components from plant residues.

## Figures and Tables

**Table 1:** Phenolic compounds contents (mg/g) in *M. communis* leaf ethanolic extracts control (EE_MC) and by-product (EE_MB).

Phenolic Compound	RT (mn)	Content (mg/g)	
		EE_MC ^1^	EE_MB ^2^
Gallic acid	7.48	5.75	12.70
Catechin	14.78	50.00	77.40
Syringic acid	17.23	0.40	4.45
p-Coumaric acid	19.98	25.20	15.70
Luteolin 7-O-glucoside	21.12	11.00	26.00
Kaempferol	26.00	0.90	0.30

^1^ Ethanolic extract obtained from *M. communis* leaf control. ^2^ Ethanolic extract obtained from *M. communis* leaf by-products.

**Table 2:** IC_50_ values (µg/mL) obtained from DPPH, ABTS and FRAP tests of *M. communis* leaf ethanolic extracts control (EE_MC) and by-product (EE_MB).

	ABTS Test IC_50_ (μg/mL)	DPPH Test IC_50_ (μg/mL)	FRAP Test IC_50_ (μg/mL)
EE_MC ^1^	4.00 ± 0.1 ^b^	12.00 ± 0.1 ^b^	117.00 ± 0.4 ^b^
EE_MB ^2^	2.00 ± 0.5 ^c^	13.00 ± 0.8 ^a^	163.00 ± 0.6 ^a^
Ascorbic acid	16.00 ± 0.3 ^a^	5.00 ± 0.2 ^c^	92.00 ± 0.3 ^c^

^1^ Ethanolic extract obtained from *M. communis* leaf control. ^2^ Ethanolic extract obtained from *M. communis* leaf by-products The mean IC_50_ values (µg/mL) obtained from DPPH, ABTS and FRAP tests of samples followed by different lowercase superscript letters are significantly different (*p* < 0.05).

**Table 3:** Inhibition zone diameter (mm) of *M. communis* leaf ethanolic extracts control (EE_MC) and by-product (EE_MB).

Bacterial Strain	*M. communis* EEs	Inhibition Zone Diameter (mm)	Sensitivity
*Staphyloccocus aureus* ATCC 25923	EE_MC ^1^	20 ± 0.6 ^a^	+++ ^3^
	EE_MB ^2^	20 ± 0.6 ^a^	+++ ^3^
*Salmonella entritidis* ATCC 13076	EE_MC ^1^	18 ± 1 ^a^	++ ^4^
	EE_MB ^2^	16 ± 1 ^b^	++ ^4^
*Aeromonas hydrophyla* ATCC 49140	EE_MC ^1^	21 ± 1 ^a^	+++ ^3^
	EE_MB ^2^	12 ± 1 ^b^	+ ^5^
*Listeria monocytogenes* ATCC 19115	EE_MC ^1^	20 ± 1 ^a^	+++ ^3^
	EE_MB ^2^	17 ± 1 ^b^	++ ^4^

^1^ Ethanolic extract obtained from *M. communis* leaf control. ^2^ Ethanolic extract obtained from *M. communis* leaf by-products. ^3^ Highly inhibitory activity (21 mm ≤ diameter ≤ 29 mm). ^4^ Mildly inhibitory activity (16 mm ≤ diameter ≤ 20 mm). ^5^ Slightly inhibitory activity (11 mm ≤ diameter ≤ 15 mm). The mean inhibition zone diameter (mm) of samples followed by different lowercase superscript letters are significantly different (*p* < 0.05).

## Data Availability

The data presented in this study are available on request from the corresponding author. The data are not publicly available due to privacy restrictions.
